# Value of transfusion independence in severe aplastic anemia from patients’ perspectives – a discrete choice experiment

**DOI:** 10.1186/s41687-018-0032-y

**Published:** 2018-03-01

**Authors:** A. Simon Pickard, Lynn Huynh, Jasmina I. Ivanova, Todor Totev, Sophia Graham, Axel C. Mühlbacher, Anuja Roy, Mei Sheng Duh

**Affiliations:** 1 Second City Outcomes Research, Chicago, IL USA; 2 Analysis Group, Inc., 111 Huntington Avenue, 14th Floor, Boston, MA 02199 USA; 3 Analysis Group, Inc., New York, NY USA; 4 Analysis Group, Inc., Menlo Park, CA USA; 5 IGM Institute Health Economics and Health Care Management at Hochschule Neubrandenburg, Neubrandenburg, Germany; 6 Novartis Pharmaceuticals Corporation, East Hanover, NJ USA

**Keywords:** Severe aplastic anemia, Transfusions, Preference, Discrete choice experiment, Bone marrow failure

## Abstract

**Background:**

Aplastic anemia is a rare, serious blood disorder due to bone marrow failure to produce blood cells. Transfusions are used to reduce risk of bleeding, infection and relieve anemia symptoms. In severe patients, transfusions may be required more than once/week. It is unclear from the patient perspective the impact that transfusions have on quality of life. This study aimed to elicit patient preferences for attributes associated with severe aplastic anemia (SAA) treatment, including transfusion independence.

**Methods:**

An online discrete choice experiment (DCE) was conducted among patients with SAA who experienced insufficient response to immunosuppressive therapy and transfusion dependence for ≥3 months in the past 2 years. Recruitment occurred through the Aplastic Anemia and Myelodysplastic Syndromes International Foundation and referrals from clinical sites in the US and France. Respondents chose between hypothetical treatment pairs characterized by a common set of attributes: transfusions frequency, fatigue, risk of infection, and risk of serious bleeding. Conditional logit model with effects coding was used to estimate part-worth utilities for different attribute levels and the relative importance of each attribute. Predicted utility scores for transfusion frequency levels were reported.

**Results:**

Thirty patients completed the survey. Most were age ≥ 40 years (73.3%), female (70.0%), and from the US (86.7%). 33.3% underwent bone marrow transplant; 36.7% received iron chelation therapy. Patients largely agreed that transfusion independence would result in less burden on time and costs, greater control and quality of life, less fatigue (86.7% noted each) and less scheduling around medical appointments (83.3%). The DCE found highest relative importance for risk of bleeding (0.30), followed by risk of infection (0.28), fatigue (0.23), and frequency of transfusions (0.20). More frequent transfusions resulted in lower utility, particularly when increasing monthly transfusions frequency from 4 (0.57) to 8 (0.35).

**Conclusions:**

Our study showed that higher utility was associated with fewer transfusions in SAA patients with insufficient response to immunosuppressive therapy. While risk of bleeding, risk of infection, and fatigue were more important for patient treatment preferences, frequency of transfusions was also important.

## Background

Aplastic anemia is a rare blood disorder where the bone marrow does not make enough red blood cells, white blood cells and platelets for the body [18]. Aplastic anemia can affect people of any age and either gender, and may appear suddenly or slowly emerge over a long period of time [1]. In the United States, about 600–900 people are diagnosed each year, according to the Aplastic Anemia and MDS International Foundation [1]. Patients are considered to have severe aplastic anemia (SAA) if two of the following three conditions are present: absolute neutrophil count (ANC) is less than 500 cells per microliter, reticulocyte count is less than 20,000 per microliter, or platelet count is less than 20,000 per microliter [2].

Survival has dramatically improved in SAA because of advances in hematopoietic stem cell transplantation (HSCT), pharmacotherapy, and supportive care in recent decades [27]. According to guidelines for management of aplastic anemia, allogeneic HSCT from a matched sibling donor is the initial treatment of choice for newly diagnosed patients if they have severe or very severe aplastic anemia, are < 40 years old and a matched sibling donor [13, 15]. Immunosuppressive therapy (IST) is recommended for (i) patients with non-severe aplastic anemia who are transfusion dependent (ii) patients with severe or very severe disease who are > 40 years old and (iii) younger patients with severe or very severe disease who do not have a matched sibling donor. The standard immunosuppressive regimen is a combination of antithymocyte globulin (ATG) and cyclosporine. Approximately 30% of patients with SAA are refractory to IST and suffer from persistent, severe cytopenia and deficits in hematopoietic stem cells and progenitor cells [15].

For patients who have SAA refractory to IST or are ineligible for HSCT, a main goal of patient management is to alleviate the SAA symptoms, such as chronic fatigue due to low red blood cells (RBC), risk of infection due to low white blood cells (WBC), and risk of bleeding due to low platelet counts. Pancytopenia results when there are low levels of RBCs, WBCs, and platelets. Alleviating SAA symptoms is typically achieved with frequent blood transfusions. In SAA patients, transfusions may be required more than once per week [33]. The reliance on blood transfusions has been found to impact quality of life due to the time required, inconvenience, and the risk of side effects in patients with SAA as reported through patient focus group discussions. While there is no literature on the impact of transfusions on quality of life in patients with aplastic anemia, the impact of frequent transfusions on quality of life has been observed in patients with myelodysplastic syndrome (MDS) who experienced anemia [3, 14]. In particular, one cross-sectional study showed that the number of transfusions per month was inversely correlated with health related quality of life (HRQOL) in MDS patients [19].

With the introduction of an oral thrombopoietin mimetic treatment, eltrombopag, for SAA patients who experienced insufficient response to IST, the reliance on blood transfusions may be reduced and some patients may achieve transfusion independence. From clinical trial study results, a reduction in the requirement for transfusions was observed among patients who received eltrombopag and have a hematologic response [5]. In patients with myelodysplastic syndromes, transfusion independence has shown to improve quality of life [19]. Prior to this study, a qualitative study was performed and a list of key attributes and levels important to patients’ value of transfusion independence were proposed by clinicians and patients. However, the qualitative study did not offer the dimension of ranking these attributes in terms of importance. Given the gap in the literature on the value of transfusion independence among patients with SAA who have had insufficient response to IST, this study aims to elicit patient preferences for attributes associated with treatment of SAA, including transfusion independence. Furthermore, understanding attributes that lead to lower utility may have important implications to the treatment paradigm for patients with severe aplastic anemia given eltrombopag has been suggested to be administered with immunosuppression therapy as first-line treatment [33].

## Methods

### Study design

This study was designed in two phases: Phase I was a qualitative study to identify important aspects of SAA treatment and to develop the survey questionnaire and Phase II involved the conduct of a one-time, non-interventional anonymous online survey study among adult patients with SAA to elicit patient preferences for attributes associated with treatment of SAA, including transfusion independence. All study materials were approved by the Ethical and Independent Review Services (E&I) Institutional Review Board (E&I study number 15128–01). No compensation was offered and informed consent was obtained from all patients who participated in the survey study.

Phase II included an interactive discrete choice experiment (DCE). The DCE was selected because it is a choice-based survey research methodology that captures patient’s value for specific characteristics of treatment and patient’s willingness to accept tradeoffs between characteristics [12, 23]. It is an established methodology for eliciting patient preferences in regards to treatment characteristics [17, 25, 34]. A patient survey using discrete choice experiment design presents patients with a series of hypothetical choice scenarios between two treatments, characterized by the same set of attributes but where one or more of the attributes have different levels, and requests patients to make a choice between the two options. By presenting patients with a DCE, the study is able to elicit patient preferences without directly asking the patient about their preference, which is indicative of patients’ actual choice regarding potential treatment [9, 23].

The survey questionnaire was developed based on findings from Phase I of this study, which included seven one-time, one-hour long, phone-based focus group discussions (Phase I focus groups). A literature review was performed to assist with the development of the moderator’s guide for the Phase I focus group discussions. Two separate moderator’s guides were developed – one for the clinicians and one for the patients. A list of potential factors that may influence a patient’s value of transfusion independence was summarized from the literature review. Participants were recruited through the physician referrals in the United States and France. The Phase I focus groups aimed to identify and refine a list of attributes that were important to the management and treatment of SAA. Focus groups in the United States and France were conducted separately with physicians (*N* = 3), a nurse (*N* = 1), and patients (*N* = 9) to discuss the illness experience and the treatment and management of SAA, followed by discussion of the value of being transfusion-independent from the patient perspective. Providers and patients gave feedback on the comprehensiveness of the attributes. The most meaningful treatment characteristics to patients were discussed, including frequency of transfusions, symptoms, risks, convenience, and costs. The attributes were rated in terms of importance on a scale from 1 (least important) to 10 (most important) from the perspective of a patient with severe aplastic anemia who is considering treatment/management options. They were asked to focus on the importance of each attribute prior to transfusion independence. The self-rating of each attribute was transformed into a rank based on relative importance and the ratings were reviewed with each respondent. Respondents were given an opportunity to reconsider their ranking and then confirm the ranking of each attribute. Providers were asked to rank and then rate the importance of each attribute from a patient perspective, and provided input on the appropriate wording for the levels for each attribute that would constitute meaningful categories.

In Phase II, the online survey consisted of three parts: 1) study qualification questions and questions collecting general patient characteristics and medical condition, 2) questions aiming to assess potential impacts of achieving transfusion independence on different aspects of patients’ well-being relative to being transfusion dependent, and improvement in quality of life with a new therapy using 5-point Likert-scale rating questions, adapted from the “MDS Patient Quality of Life Survey” that was available online and has been published [29], and 3) an interactive discrete choice experiment (DCE) designed to capture patients’ willingness to accept tradeoffs between hypothetical treatment characteristics. The online survey was designed to be completed in English or French. The survey was designed in English and translated into French based on the efforts of three professional linguists, each chosen based on their knowledge of the language, the subject area, and the target audience. Once the translation was completed, an editor carefully reviewed and refined the document to ensure that it read as if it were originally written in French. A proofreader then provided quality control, checking the translation against the original to verify that it appropriately represented the source.

The key attributes and levels for the DCE experiment, selected based on the findings from the Phase I focus groups, included the following: risk of serious bleeding (low: platelet count > 50,000, moderate: platelet count 10,000–50,000, high: platelet count < 10,000), risk of infection (low: ANC > 1000 cells/mm^3^, moderate: ANC 500–1000 cells/mm^3^, high: ANC < 500 cells/mm^3^), fatigue (none, moderate, severe), and frequency of transfusion (none, 4 per month, 8 per month). These attributes were recommended based on the independence of the attributes relevant to the management of SAA (relative to other attributes initially considered such as convenience, which converged as a concept with frequency of transfusion), importance as rated by clinicians and patients and for their consistent relevance in focus groups with clinicians and patients from the US and France.

**Fig. 1:**
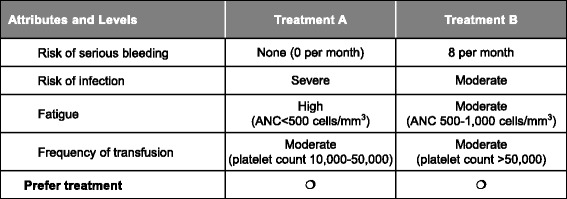
Example of comparison card. Abbreviaton: ANC, Absolute Neutrophil Count

In the questionnaire, patients were asked to choose between pairs of hypothetical treatments characterized by a common set of attributes in order to estimate values for the attributes. To reduce the burden on the patient in responding to multiple hypothetical treatment comparisons, each patient reviewed 12 comparison cards. An opt-out option was not included, as receiving no treatment does not mirror the real-life experience of patients with SAA. The DCE design was generated using Sawtooth Software’s Balanced Overlap method [26]. This method minimizes the frequency respondents will have to compare the same levels throughout the tasks, but allows that in a given task both treatment options can compare the same level for an attribute, which results in more plausible choice options. All presented treatment profiles with different levels of attributes are plausible (e.g., low level of transfusions and low levels of symptoms/risks, high levels of transfusions and low levels of symptoms/risks, etc.). Versions of the design that included a dominant comparison, where one treatment was better in all attributes than the alternative, were removed. The final DCE design consisted of 20 versions, each with 12 comparison cards that compared two treatments. An example of the comparison card is shown in Fig. 1.

### Data source

Patients who participated in this study were recruited through announcements in the newsletter of the Aplastic Anemia and Myelodysplastic Syndromes International Foundation and referrals from clinical sites in the US and France. Thirty patients qualified for the study and completed the online survey anonymously between February 2, 2016 and April 15, 2016. Patients did not receive a monetary incentive for completion of the survey.

### Study population

Patients who completed the survey were screened to meet the following criteria to qualify for this study: age 18 years or older, diagnosed with SAA by a doctor or healthcare provider, prescribed IST with antithymocyte globulin but had an insufficient response, required blood transfusions at least monthly for a period of at least three months over the previous two years, and not currently pregnant.

### Outcomes and statistical analysis

Demographic and SAA-related characteristics questions (country, age, gender, race [asked in the USA only], education level, employment status, health insurance coverage, history of treating SAA with drugs, transfusions, and iron chelation therapy) consisting of categorical variables were analyzed using descriptive statistics, and the number and proportion of patients were reported. Questions in which the patient rated their current condition and characteristics related to being transfusion independent were also analyzed using descriptive statistics, and the number and proportion of patients with top-two box (most favorable) Likert-scale ratings were reported.

For the DCE, a conditional logit model was used to estimate part-worth utilities for different attribute levels and assess the relative importance of each attribute [12]. Part-worth utility is a computed preference weight for each attribute and its respective levels. A negative part-worth utility indicates less desired levels of the attributes, whereas a positive part-worth utility indicates more desired levels of the attributes. In our model, attribute levels were effect-coded, whereby omitted categories were coded as − 1, so the part-worth utility estimate of the omitted category for each attribute is the negative sum of the part-worth utility estimates for the included categories. For example, if the treatment alternative had the level of the omitted attribute level (e.g., high risk of serious bleeding) then the dummy variables of the attribute levels included in the model (e.g., low risk of serious bleeding and moderate risk of serious bleeding) had a value of − 1. With effects coding, part-worth utility estimates represent the deviation of the mean of each attribute level from the overall mean/the model intercept. The model assumes that an individual’s utility function, or choice, can be defined by the level’s part-worths, or coefficient estimates [4, 8]. An individual’s probability of choosing a given treatment when considering two treatment options can be expressed as shown in eq. 1 [22].

1 $$ \mathrm{Probability}\ \mathrm{of}\ {\mathrm{treatment}\ \mathrm{choice}}_{Treat ment\ X}=\frac{e^{\left({\mathrm{Total}\ \mathrm{utility}}_{Treat ment\ X}\right)}}{e^{\left({\mathrm{Total}\ \mathrm{utility}}_{Treat ment\ X}\right)}+{e}^{\left({\mathrm{Total}\ \mathrm{utility}}_{Treat\mathrm{m} ent\ Y}\right)}} $$

Attribute relative importance and level ranking are made based on comparisons of part-worths [21]. An attribute with a larger difference between its lowest level part-worth and its highest level part-worth is considered to have a greater relative importance in the patient’s choice [4, 21]. Within a given attribute, the relative differences between the levels correspond to the relative respondent preferences. The relative contribution of each treatment attribute to patient treatment preferences was calculated based on the coefficient range for each attribute as a proportion of the summed range of coefficients across all attributes as shown in eq. 2.

2 $$ {\mathrm{Relative}\ \mathrm{contribution}}_{Attribute}\%=\frac{100\times \left(\mathrm{Maximum}\ \mathrm{part}-{\mathrm{worth}}_{Attribute}-\mathrm{Maximum}\ \mathrm{part}-{\mathrm{worth}}_{Attribute}\right)}{\sum \left(\mathrm{Maximum}\ \mathrm{part}-{\mathrm{worth}}_{Attribute}-\mathrm{Maximum}\ \mathrm{part}-{\mathrm{worth}}_{Attribute}\right)} $$

Predicted utility scores associated with different levels of transfusion frequency were also estimated to understand the effect of transfusion frequency on choice. The predicted utility scores with different levels of transfusion frequency were estimated using the parameter estimates from the conditional logit model and out of sample prediction of utility scores assuming scenarios where all treatment alternatives required 0, 4, and 8 transfusions per month, respectively.

All analyses were performed using SAS 9.4. DCE analyses were validated using Stata 14.1.

## Results

### Patient characteristics

A total of 30 patients completed the anonymous survey (Table 1). Most of the patients who completed the survey were from the United States (26 [86.7%] patients), and the remaining four (13.3%) came from the United Kingdom, France, Canada, and Australia. Most patients were age ≥ 40 years (73.3%) and 33.3% were over 65. The majority (70.0%) of patients were female. One-third (33.3%) of the patients were employed full-time, part-time or self-employed; 30.0% were retired, 20.0% were disabled, and the remaining 13.3% self-identified as homemaker or unemployed. All patients had coverage for medical care (100.0%) and most had coverage for medication (96.7%) at the time of taking the survey.

**Table 1:** Respondent characteristics

	All Respondents (*N* = 30)	
Gender, N (%)		
Male	9	(30.0%)
Female	21	(70.0%)
Age, N (%)		
18–49	10	(33.3%)
50–64	10	(33.3%)
65+	10	(33.3%)
Country of Residence, N (%)		
USA	26	(86.7%)
UK	1	(3.3%)
France	1	(3.3%)
Australia	1	(3.3%)
Canada	1	(3.3%)
Education,^a^ N (%)		
High school degree only	3	(10.0%)
Some college, no degree	6	(20.0%)
Associate’s degree (e.g., 2-year college degree)	2	(6.7%)
Bachelor’s degree	9	(30.0%)
Master’s degree or higher	10	(33.3%)
Employment, N (%)		
Retired	9	(30.0%)
Disabled	6	(20.0%)
Employed full-time	5	(16.7%)
Employed part-time	3	(10.0%)
Homemaker	3	(10.0%)
Self-employed	2	(6.7%)
Unemployed, but looking for work	1	(3.3%)
Unemployed, not looking for work	1	(3.3%)

^a^Foreign education systems were mapped into the American education system

Fifty percent of patients were diagnosed with SAA in the previous 2 years, 26.7% were diagnosed 3 to 4 years ago, and 23.3% were diagnosed 5 or more years ago (Table 2). Among the symptoms and complications patients have experienced since their diagnosis, 86.7% reported fatigue that interfered with daily activities like work and school, 53.3% experienced infections requiring hospitalization, and 53.3% experienced bleeding.

**Table 2:** Diagnosis and treatment

	All Respondents (*N* = 30)	
First Diagnosed with SAA, N (%)		
2 or fewer years ago	15	(50.0%)
3 to 4 years ago	8	(26.7%)
5 or more years ago	7	(23.3%)
Symptoms and Complications,^a^ N (%)		
Fatigue (interferes with daily activities like work and school)	26	(86.7%)
Infections requiring hospitalization	16	(53.3%)
Bleeding	16	(53.3%)
Number of Treatments Received after SAA Diagnosis, N (%)		
1–3	13	(43.3%)
4–5	10	(33.3%)
6 or more	7	(23.3%)
Types of Treatments Received after SAA Diagnosis,^a^ N (%)		
Immunosuppressive therapies	29	(96.7%)
Steroids	24	(80.0%)
Targeted therapy	17	(56.7%)
Antibiotics	17	(56.7%)
Antifungals	14	(46.7%)
Bone marrow transplant	10	(33.3%)
Erythropoiesis-stimulating agents/growth factors	9	(30.0%)
Red Blood Cell or Whole Blood Transfusions Frequency, N (%)		
Have not received red blood cell transfusions in the past 3 months	16	(53.3%)
Once every 31–90 days	4	(13.3%)
Once every 15–30 days	5	(16.7%)
Once every 8–14 days	5	(16.7%)
Once per week	0	(0.0%)
More than once per week	0	(0.0%)
Platelet Transfusions Frequency, N (%)		
Have not received platelet transfusions in the past 3 months	21	(70.0%)
Once every 31–90 days	3	(10.0%)
Once every 15–30 days	1	(3.3%)
Once every 8–14 days	2	(6.7%)
Once per week	3	(10.0%)
More than once per week	0	(0.0%)
Received Iron Chelation Therapy, N (%)	11	(36.7%)

^a^Respondents were allowed to select multiple values for symptoms and complications, and treatments received, thus counts and percentages may sum to more than the total N or 100%

### Treatment history and experience with transfusion

The most common treatment patients received was IST (96.7%), followed by steroids (80.0%), targeted therapy (56.7%), and antibiotics (56.7%) (Table 2). Fewer than half of the patients were treated with antifungals (46.7%) or erythropoiesis-stimulating agents/growth factors (30.0%), or received a bone marrow transplant (33.3%). In the 3 months before the time of survey completion, 53.3% of patients had not received red blood cell or whole blood transfusions, while 13.3% had received transfusions once every 31–90 days, 16.7% had received transfusions once every 15–30 days, and 16.7% had received transfusions once every 8–14 days. In regards to platelet transfusion in the past 3 months, 70.0% answered they had not received white blood cell transfusions in the past 3 months, while 10.0% had received transfusions once every 31–90 days, 10.0% had received transfusions once or twice per month, and 10.0% had received transfusions once per week. 36.7% of patients had received iron chelation therapy in the past. When asked to rate agreement on statements related to their condition in the past week, over half of the patients (60.0%) reported feeling fatigued, 46.7% agreed with being frustrated by not being able to do their usual activities, and 43.3% reported bruising easily.

### Opinions about transfusion independence

Most patients agreed that achieving transfusion independence would result in less burden in terms of time and costs, greater quality of life and life enjoyment, less fatigue, and greater control despite their health condition (each noted by 86.7% of patients) when asked to rate agreement on statements about their opinion on the impact of achieving transfusion independence on their well-being (not shown). Patients most often noted the following as important when they were asked to rate the importance of several ways to improve their quality of life with a new therapy: less frequent transfusions, less fatigue, and better emotional well-being (each endorsed by 96.7% of patients). Lower risk of infection and lower risk of serious bleeding were also of high importance to patients (each noted by 90.0% of patients).

### Discrete choice experiment analysis

**Fig. 2:**
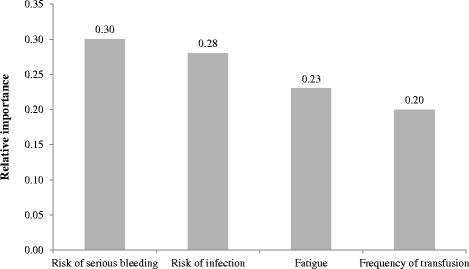
Relative contribution of each treatment attribute to patient treatment preferences (*N* = 30)^a^. Note: a. The relative contribution of each attribute represents the relative importance each attribute has on treatment preferences. The relative importance of each attribute was calculated as the range of part-worths for the attribute divided by the sum of part-worth ranges for all attributes

**Fig. 3:**
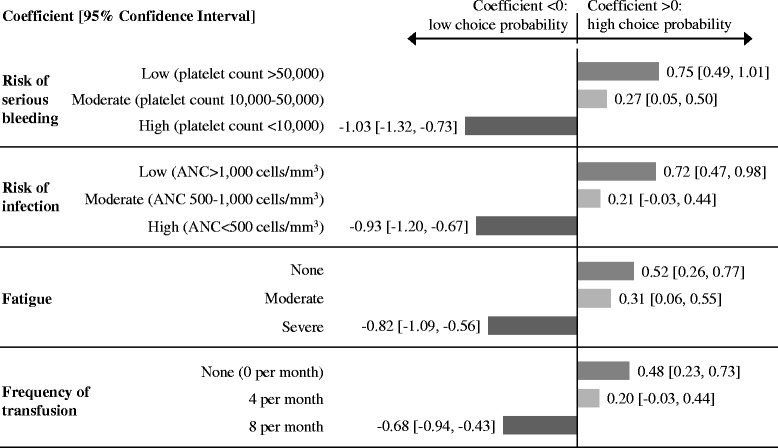
Effects of attribute levels on health state preference: part-worth utilities (*N* = 30). Abbreviation: ANC, Absolute Neutrophil Count

**Fig. 4:**
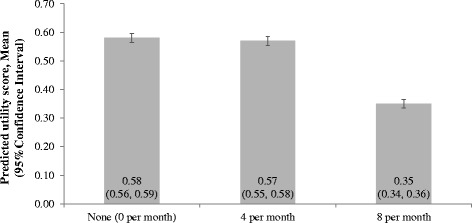
Predicted utility with different frequency of transfusion (*N* = 30)^a^. Abbreviation: CI, Confidence Interval. Note: a. The predicted utility score was estimated using estimates from a generalized linear model with logit link and binomial distribution for the error term and assuming scenarios where all treatment alternatives required 0, 4, and 8 transfusions per month, respectively. Analyses were performed using SAS 9.4

Based on the logit model, risk of bleeding was found to be the most important factor in patient treatment preferences with a relative importance of 0.30, followed by risk of infection (0.28), fatigue (0.23), and frequency of transfusions (0.20) (Fig. 2; no statistical comparison of relative importance between attributes was conducted). Figure 3 illustrates the relationship between the probability of selection (also referred to as part-worth utility or preference weight) and the attribute levels. A positive coefficient indicates high choice probability, whereas a negative coefficient indicates low choice probability. From Fig. 3, levels of “low” and “moderate” risk of serious bleeding and risk of infection, levels of “none” and “moderate” fatigue, and “none” for frequency of transfusion had positive coefficients which showed there was a high probability of selection. Levels of “high” risk of serious bleeding and risk of infection, “severe” fatigue, and “8 per month” transfusions resulted in negative coefficients indicating low probability of selection. To illustrate this concept, for the attribute frequency of transfusion, receiving more transfusions per month was associated with low probability in treatment selection. In particular, a large utility decrease was found for increasing the frequency of transfusions from 4 to 8 per month: the part-worth utility decreased from 0.48 associated with 0 transfusions per month to 0.20 associated with 4 transfusions per month, to − 0.68 associated with 8 transfusions per month (Fig. 3). This showed that receiving 8 transfusions per month had a negative influence on patient preferences, while no transfusions per month had high probability of selection. The mean utility score estimated by only varying the frequency of transfusion and keeping all other attributes constant was 0.58 associated with 0 transfusions per month, 0.57 associated with 4 transfusions per month, and 0.35 associated with 8 transfusions per month (Fig. 4). Prior to performing the data analysis, responses were checked for straight-lining. No such survey completion was found in the data.

Similarly to the frequency of transfusions, larger declines in part-worth utilities were estimated between the moderate and high/severe levels of risk of serious bleeding, risk of infection, and fatigue, compared to the decrease in utilities between the low/no and moderate levels (Fig. 3). All evaluated attribute levels had statistically significant part-worth utility coefficients at the 95% confidence level, except for moderate risk of infection and 4 transfusions per month.

To describe potential heterogeneity of preferences, the following descriptive analyses were conducted. Conditional logit models and relative importance of attributes to treatment preferences were estimated for patient subgroups by age (age 18–49, *n* = 10; age 50 and older, *n* = 20), years since first diagnosis with SAA (≤2 years ago, *n* = 15; 3 or more years ago, n = 15), experiencing bleeding since diagnosis (Yes, *n* = 16; No, *n* = 14), experiencing infection requiring hospitalization since diagnosis (Yes, *n* = 16; No, *n* = 14); and having any transfusions in the past 3 months (Yes, *n* = 14; No, *n* = 16). No statistical test of heterogeneity was performed.

Risk of bleeding and risk of infection had the highest relative contributions to treatment preferences but depending on the subgroup, their order in the treatment preferences differed. For example, risk of bleeding had numerically higher relative contribution to treatment preferences among patients who were younger (relative contribution 0.36 for age 18–49 years vs. 0.27 for patients 50 and older); were diagnosed with SAA more recently (relative contribution 0.33 for SAA diagnosis ≤2 years ago vs. 0.28 for SAA diagnosis 3 or more years ago), experienced bleeding since diagnosis (relative contribution 0.34 among patients who experienced bleeding vs. 0.26 among patients who did not), and experienced infection requiring hospitalization (relative contribution 0.35 among patients who experienced infection vs. 0.26 among patients who did not). Risk of infection had numerically higher relative contribution to treatment preferences among patients who were older (relative contribution 0.21 for age 18–49 years vs. 0.32 for patients 50 and older), were diagnosed with SAA longer ago (relative contribution 0.30 for SAA diagnosis ≤2 years ago vs. 0.26 for SAA diagnosis 3 or more years ago), did not experience bleeding since diagnosis (relative contribution 0.23 among patients who experienced bleeding vs. 0.33 among patients who did not), and did not experience infection since diagnosis (relative contribution 0.25 among patients who experienced infection vs. 0.30 among patients who did not). While the result that risk of bleeding had higher relative contribution than infection among patients who experienced infection may seem counter-intuitive, 10 out of 16 patients (62.5%) who had infection also experienced bleeding. Similar to the overall analysis, fatigue and frequency of transfusion generally had lower but meaningful relative contribution (fatigue relative contribution ranged from 0.21 among patients diagnosed 3 or more years ago and patients who did not experience bleeding to 0.24 among patients who experienced bleeding). Among patients who did not have any transfusions in the past 3 months (proxy for transfusion independence), the relative contribution of the frequency of transfusions (0.25) was numerically higher than among patients who had transfusions in the past 3 months (0.14). While risk of bleeding had the highest relative contribution to preferences among patients without transfusion in the past 3 months (0.28), frequency of transfusion had a relative contribution, which was similar to that of risk of infection (0.25) and higher than that for fatigue (0.22).

## Discussion

This study provided primary research to help to understand the value of transfusion independence among patients with SAA, a rare condition. The study found that among patients with SAA with insufficient response to IST, the estimated utility was higher with fewer transfusions. While attributes such as risk of bleeding, risk of infection, and fatigue were more important for patient treatment preferences, frequency of transfusions was also important for treatment preferences. Over 80% of patients agreed that achieving transfusion independence would result in less burden on time, less need to arrange life around medical appointments, improved quality of life, less fatigue, and feeling in control despite their health condition.

Approximately 50% of patients who took this survey reported feeling fatigued, being frustrated by not being able to do their usual activities, and bruising easily over the past 7 days. A majority of the patients (96.7%) found it important for a new therapy to result in less frequent transfusions, less fatigue, and better emotional well-being. The DCE analysis helped to clarify that while patients may be less sensitive to the difference between transfusion independence (receiving 0 transfusions per month) and receiving up to 4 transfusions per month, they have a strong preference against increasing the frequency of transfusions to 8 per month. This is in line with findings from focus group discussions about patients tending to get used to the administration of blood transfusions, and worrying more about risk of infection, risk of bleeding and fatigue, which have a much stronger impact on their quality of life. Focus group participants explained that transfusions typically take several hours per procedure and require traveling to the hospital, which becomes very burdensome if required frequently.

Several published studies discuss the clinical impact of transfusion dependence among SAA patients, mainly the high risk of iron overload caused by red blood cell transfusions, which is treated with iron chelation therapy [11, 20, 31]. Iron overload can lead to multiple organ failure and is generally associated with an increase in morbidity and mortality [20, 31]. This study was the first to provide primary research to help to understand the value of achieving transfusion independence from SAA patients’ perspective.

Due to limited published research on SAA patients, the results found in this study could not be compared to findings from other studies in SAA. However, the similarity between SAA and MDS as bone marrow failure states [24] allows for comparisons to studies in the MDS population. The results of this study are consistent with previous studies of MDS patients. Multiple studies have reported on the high frequency of manifestation and the detrimental effect that fatigue poses on MDS patients’ quality of life, which is controlled with RBC transfusions [10, 14, 16, 28]. In our study, fatigue was the most frequently mentioned complaint, reported by 60.0% of the patients with SAA. Almost all of our survey respondents (96.7%) found it important for a new therapy to improve quality of life with less fatigue. The DCE analysis showed that fatigue was an important factor in patient treatment preference, with a relative importance coefficient of 0.23 and a very strong disutility of − 0.82 for severe fatigue. In addition, four out of the nine patients (44.4%) who participated in the Phase I focus group discussion noted that they had to leave their employment due to fatigue.

Other studies concluded that transfusions inconvenienced MDS patients due to time required for each transfusion and the economic burden of the cost of each transfusion [3, 6, 7, 14]. A 2012 analysis of Medicare claims data found a three-fold increase in 3-year mean cumulative Medicare costs for transfused patients with MDS compared to non-transfused patients with MDS ($88,824 vs. $29,519, *P* < 0.001) [6]. The transfusion experience was similar for SAA patients from our study. Eighty-seven percent of patients with SAA who completed the survey noted that achieving transfusion independence may result in a smaller burden on time and monetary cost of treatment. A survey study among patients with MDS found consistently significantly higher utility scores for transfusion independence compared to reduced transfusions (*P* < 0.001) or transfusion dependence (*P* < 0.001) using both the Visual Analogue Scale (VAS) the Time Trade-Off (TTO) methods [30]. Six percent of the MDS survey study’s respondents rated transfusion dependence as worse than being dead. Almost all patients with SAA who completed the survey (96.7%) also found less frequent transfusions important in improving life with a new therapy. The DCE estimated a part-worth utility of − 0.68 for the 8 transfusions per month level compared to 0.20 for 4 transfusions per month and 0.48 for none, which demonstrated a strong preference for fewer than 8 transfusions per month.

Finally, DCE results showed that patients had a stronger preference for a lower risk of serious bleeding and risk of infection compared to a lower frequency of transfusions. It was mentioned in the focus group discussions that both of these risks were impediments to participating in the workforce and in social activities. Patients complained of frequent nosebleeds and loss of eye sight due to bleeding in the eye and noted that platelet transfusions helped alleviate the problem. Several studies have established infections as a major cause of death among patients with aplastic anemia, whether it is due to their immunocompromised state or to being neutropenic due to chemotherapy [32, 35].

This study is subject to several limitations. Given that SAA is a rare disease, the study employed a convenience sampling strategy, which may not be fully representative of SAA patients of the general population of SAA with insufficient response to IST. Furthermore, all data, including treatment history and past experience with transfusion, were self-reported and could have been subjected to recall bias. In addition, the DCE component of this study was a hypothetical exercise designed to assess the relative importance of treatment attributes and levels. In reality, some of the presented combinations may not be observed. The study design assumed that the attributes were independent. However, to the extent that correlations between the attributes existed, the coefficients on the attributes may reflect more than the role of the particular attributes. Given the small sample size, the analysis did not assess preference interactions among the attributes. Lastly, treatment outcomes may be dependent on personal and treatment-specific factors outside of the scope of this research.

## Conclusions

In conclusion, patients with SAA with insufficient response to IST showed a strong preference for fewer transfusions per month. This has important implications for the introduction of new treatment as first line of therapy that could lead to transfusion independence in patients with SAA. Over 80% of patients reported that achieving transfusion independence would result in less burden on time, less need to arrange life around medical appointments, improved quality of life, less fatigue, and feeling in control despite their health condition.

## Abbreviations

ANC: Absolute Neutrophil Count
ATG: Antithymocyte Globulin
DCE: Discrete Choice Experiment
E&I: Ethical and Independent Review Services Institutional Review Board
HSCT: Hematopoietic Stem Cell Transplantation
IST: Immunosuppressive Therapy
MDS: Myelodysplastic Syndromes
MUD: Matched Unrelated Donor
RBC: Red Blood Cells
SAA: Severe Aplastic Anemia
US: United States
WBC: White Blood Cells
